# Alleviation of Carbon-Tetrachloride-Induced Liver Injury and Fibrosis by Betaine Supplementation in Chickens

**DOI:** 10.1155/2015/725379

**Published:** 2015-09-27

**Authors:** Meng-Tsz Tsai, Ching-Yi Chen, Yu-Hui Pan, Siou-Huei Wang, Harry J. Mersmann, Shih-Torng Ding

**Affiliations:** ^1^Department of Animal Science and Technology, National Taiwan University, No. 50, Lane 155, Section 3, Keelung Road, Taipei 106, Taiwan; ^2^Institute of Biotechnology, National Taiwan University, No. 81, Chang-Xing Street, Taipei 106, Taiwan

## Abstract

Betaine is a food component with well-reported hepatoprotection effects. However, the effects and mechanisms of betaine on liver fibrosis development are still insufficient. Because metabolic functions of chicken and human liver is similar, we established a chicken model with carbon Tetrachloride- (CCl_4_-) induced fibrosis for studying antifibrotic effect of betaine *in vivo* and *in vitro*. Two-week-old male chicks were supplemented with betaine (1%, w/v) in drinking water for 2 weeks prior to the initiation of CCl_4_ treatment (i.p.) until sacrifice. Primary chicken hepatocytes were treated with CCl_4_ and betaine to mimic the *in vivo* supplementation. The supplementation of betaine significantly alleviated liver fibrosis development along with the inhibition of lipid peroxidation, hepatic inflammation cytokine, and transforming growth factor-*β*1 expression levels. These inhibitive effects were also accompanied with the attenuation of hepatic stellate cell activation. Furthermore, our *in vitro* studies confirmed that betaine provides antioxidant capacity for attenuating the hepatocyte necrosis by CCl_4_. Altogether, our results highlight the antioxidant ability of betaine, which alleviates CCl_4_-induced fibrogenesis process along with the suppression of hepatic stellate cells activation. Since betaine is a natural compound without toxicity, we suggest betaine can be used as a potent nutritional or therapeutic factor for reducing liver fibrosis.

## 1. Introduction

The prevalence of worldwide metabolic syndromes increases the incidence of chronic liver diseases including liver steatosis, steatohepatitis, fibrosis, and cirrhosis [1, 2]. In the progress of chronic liver diseases, liver fibrosis acts as a sign of liver damage and a factor of liver dysfunction by progressive cirrhosis in the liver [3]. Since the long periods of liver fibrosis progress, the therapies should be tolerable and safe for over decades. However, the therapeutic interventions of liver fibrosis have not been approved yet [4]. Therefore, the safe and well-tolerated natural compounds, which provide antifibrotic ability, might be an alternative choice until appropriate drug therapies become available.

Besides human, liver injury is also a common pathology in poultry, caused by many factors, such as nutrition, diseases, and toxins [5]. Furthermore, both human and chicken use liver for over 90%* de novo* lipogenesis [6, 7]. In rodents and rabbits, the adipose tissues and liver provide the equal function of* de novo* lipogenesis [8, 9]. Therefore, the chicken have been suggested and used as an animal model for human liver diseases recently [10–12]. Although several studies described that the excessive hepatic lipogenesis and lipid accumulation are the very initiator of the liver fibrosis [2], liver fibrosis can also be a result of other liver injuries.

Liver fibrosis is the scarring process that represents liver injury and the excessive accumulation of collagen, which results from inflammation and the death of hepatocytes that occurs in most types of chronic liver diseases [3]. CCl_4_-induced liver injury is a commonly used rodent model to study liver pathologies, which shows similar morphology and the biochemical characteristic of the cellular lesions with human liver diseases [13]. Once exposed to CCl_4_, the cytochrome p450 2E1 (CYP2E1) in hepatocytes could metabolize CCl_4_ into trichloromethyl radicals (CCl_3_^−^), which then induces lipid peroxidation and causes oxidative stress [14]. Numerous studies described that the elevation of cellular oxidative stress induces secretion of inflammatory cytokines, including interleukin- (IL-) 1, IL-6, and tumor necrosis factor (TNF) [15]. Furthermore, previous studies indicate that the inflammatory cytokines can regulate the secretion of transforming growth factor- (TGF-) *β*1, which is a cytokine involved in many cellular functions, including cell proliferation, differentiation, and apoptosis. Notably, TGF-*β*1 also plays a major role in the fibrogenesis process in response to liver injury, which contributes to a critical and fundamental event in hepatic fibrogenesis, known as hepatic stellate cells (HSCs) activation [16]. The activated HSCs are the mainly cell type involved in liver fibrosis, which forms scar tissue by producing collagens and extracellular matrix in response to liver damage [17, 18]. Hence, the HSCs have been suggested as a major target for the treatment of liver fibrosis.

Betaine is a naturally occurring compound found in common food, including wheat germ, bran, vegetables, and seafood [19]. Previous review indicates that the adult human takes 1.0–2.5 grams of betaine per day from dietary intake and suggests there is nontoxicity of betaine [20]. In mammalian metabolism, betaine has two major functions, acting as a major osmolyte in the brain and kidney to modulate cell volume [21, 22] and as a methyl group donor for the methionine-homocysteine cycle [23]. Besides the well-known cellular functions of betaine, previous studies have described that the exogenous betaine improves diets-induced fatty liver syndromes, cardiovascular diseases [20, 24], and against chemicals-induced liver fibrosis [25, 26]. Since the oxidative stress is a major factor in liver injury initiation [27], previous studies have suggested the hepatoprotective activity of betaine may be related to its antioxidant capacity to protect hepatocyte from free radicals [25, 28]. However, the knowledge of betaine alleviating liver fibrosis has yet to be clarified.

In the current study, we investigate the effects of betaine against liver fibrosis induced by CCl_4_ in chickens to evaluate the potential of therapy application. We also assessed the related mechanisms involved in antifibrotic effects of betaine.

## 2. Material and Methods

### 2.1. Animal and Experimental Design

**Figure 1:**
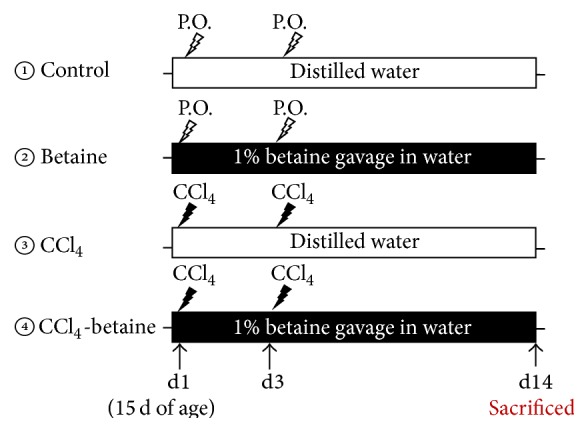
Strategy of the experimental design used to evaluate the effects of betaine on CCl_4_-induced hepatic injury in chickens. P.O.: peanut oil.

Male Hy-line chicks at 1 d of age were purchased from a local hatchery. The chickens were fed with a standard diet and water* ad libitum*. The standard chicken growing diet was based on corn, soybean meal, and beef tallow and contained 18% protein, 2.5% fat, and 6% fiber with an energy content of 2800 kcal/kg. At 15 d of age, the chickens were divided into four groups (*n* = 6): (1) control (blank) group, (2) betaine group (drinking water containing 1% (w/v) betaine), (3) CCl_4_ group, and (4) CCl_4_-betaine group (drinking water containing 1% (w/v) betaine). Groups 1 and 2 chickens were injected with peanut oil (4.0 mL/kg. BW); groups 3 and 4 chickens were injected with CCl_4_ in peanut oil at a ratio of 1 : 1, v/v (4.0 mL/kg. BW). To induce liver fibrosis, CCl_4_ (or peanut oil for groups 1 and 2) was injected [5] into the pectoral muscle on experimental d1 (15 d of age) and d3. Each group of chickens was housed in independent compartments, and the environmental temperature and relative humidity were maintained in 25°C and 70%. Betaine (Sigma-B2629, Sigma-Aldrich, St. Louis, MO, USA) was given in the drinking water to group 2 and group 4 from experimental d1 to d14. The experimental design was shown in Figure 1.

On experimental d1 and d14, blood samples were collected from the carotid artery, and ethylenediaminetetraacetic acid was used as anticoagulant. Samples were centrifuged at 2,000 ×g for 10 min to collect plasma. Chickens were killed by carbon dioxide to harvest the livers on experimental d14. A portion of the liver was fixed in 10% formaldehyde for histology analysis. Another portion of the liver was frozen in liquid nitrogen and stored at −80°C. All procedures were approved by the Institutional Animal Care and Use Committee of National Taiwan University.

### 2.2. Biochemical Analysis

Plasma was diluted with 0.9% (w/v) NaCl and analyzed for alanine aminotransferase (ALT; AL1268, RANDOX, Antrim, UK) and aspartate aminotransferase (AST; AS101, RANDOX, Antrim, UK) by colorimetric end point assays according to the manufacturer's instructions. Liver 8-hydroxy-2-deoxyguanosine (8-OHdG; MBS261211, MyBioSources, Inc., San Diego, CA, USA) and plasma dipeptidyl-peptidase 4 (DPP4; MBS023397, MyBioSources, Inc., San Diego, CA, USA) and glutathione S-transferase (GST; MBS743037, MyBioSources, Inc., San Diego, CA, USA) concentrations were measured with chicken-specific ELISA kits according to the manufacturer's instructions.

The liver malondialdehyde (MDA) assay was used to assess degree of lipid peroxidation. It was measured by thiobarbituric acid test described in the previous study [29]. The betaine concentrations in chicken liver were measured by colorimetric method, as described previously, with minor modifications [30]. In briefly, the betaine can be deposited by ammonium Reinecke salt in acidic environment (pH 1.0), the deposited-complex could dissolve in 70% acetone, and the absorbance of the colored-solutions was measured at 525 nm.

### 2.3. Histology Analysis

Livers were fixed in 10% buffered formaldehyde, embedded in paraffin, sectioned at 5 *μ*m, and stained with Masson's trichrome stain to examine the existence of liver fibrosis, that is, collagen deposition. Stained liver sections were analyzed using ImageJ software (NIH, Bethesda, MD, USA).

### 2.4. Quantitative Real-Time PCR

Total RNA was extracted from frozen liver tissue samples using TRIzol (Invitrogen Carlsbad, CA, USA). The TURBO DNA-free kit (Invitrogen Carlsbad, CA, USA) was utilized to remove the contamination of genomic DNA. mRNA was then reverse transcribed to cDNA using a High Capacity cDNA Reverse Transcription kit (Applied Biosystems, Foster City, CA, USA). Quantitative real-time PCR reactions were performed on a StepOne Plus real-time PCR System (Applied Biosystems, Foster City, CA, USA) using a DyNAmo Flash SYBR Green kit (Finnzymes, Espoo, Finland). Running conditions for real-time PCR were initial denaturation at 95°C for 7 min and denaturation at 95°C for 10 s, annealing at appropriate temperature for each pair of primers and extension for 30 s for a total of 38 cycles. Finally, there was an extension time of 30 s at 72°C. The primer sequences and annealing temperatures were listed in Table 1. Threshold cycle (Ct) values were obtained and relative gene expression was calculated using the formula (1/2)^Ct  target  gene–Ct^ *β* ^-actin^ [31]. The *β*-actin mRNA was measured in each sample by real-time quantitative PCR as a reference gene and levels of mRNA were expressed as a ratio with reference to the expression of *β*-actin.

### 2.5. Cell Isolation and Culture

Chicken hepatocytes were isolated from the one week old Hy-line male chicks [32]. Hepatocytes were plated in 6-well plates containing 2 mL Dulbecco's modified Eagle's medium (DMEM) (Invitrogen Carlsbad, CA, USA) with 10% chicken serum (Invitrogen Carlsbad, CA, USA) and 100 U penicillin/mL, 100 mg streptomycin/mL, and 1.5 *μ*g/mL amphotericin B. Cells were cultured at 37°C in air containing 5% CO_2_. Each well contained 1 × 10^5^ cells; incubations were continued until the cells reached 80 to 90% confluence. Then, the medium was removed and replaced with serum-free DMEM for 24 h. After the serum-free period, primary hepatocytes were treated with or without 5 mM CCl_4_ for 20 h to induce hepatocyte necrosis, and different concentrations of betaine (0, 5, 10, or 20 mM) were cotreated at the same time to test the betaine as an antifibrosis agent. After incubation, cells and medium were harvested for analysis.

### 2.6. Cell Survival

After 20 h exposure to betaine (0, 5, 10, or 20 mM) ± CCl_4_ (0 or 5 mM) to induce cell necrosis in chicken primary hepatocytes, cell viability was measured by using the tetrazolium, 3-(4,5-dimethylthiazol-2-yl)-2,5-diphenyltetrazolium bromide (MTT) (M5655, Sigma-Aldrich, St. Louis, MO, USA) assay [33].

### 2.7. Statistical Analysis

All results were expressed as means ± SEM. Statistical analysis was by two-way ANOVA, and means from groups with a significant ANOVA were separated using Tukey's test for statistical difference. Differences between treatments were considered to be statistically significant at *P* ≤ 0.05.

## 3. Results

### 3.1. General Findings

The general findings of the chicken at the age of d14 are shown in Table 2. No significant differences were observed in the body weight between control, betaine, and CCl_4_ groups. Only CCl_4_-betaine group had significant lower body weight than control group. In the relative liver weights, the CCl_4_-betaine group was significantly increased in comparison with other groups. To evaluate the effect of betaine on CCl_4_-induced liver injury, plasma AST and ALT were measured on d14. Notably, no significant differences in the plasma AST and ALT levels between each group were observed. The no changes of plasma AST and ALT levels are in agreement with previous studies that plasma AST and ALT are inefficient liver injury biomarkers in poultry [34, 35].

### 3.2. Effects of Betaine on Liver Fibrosis Development

**Figure 2:**
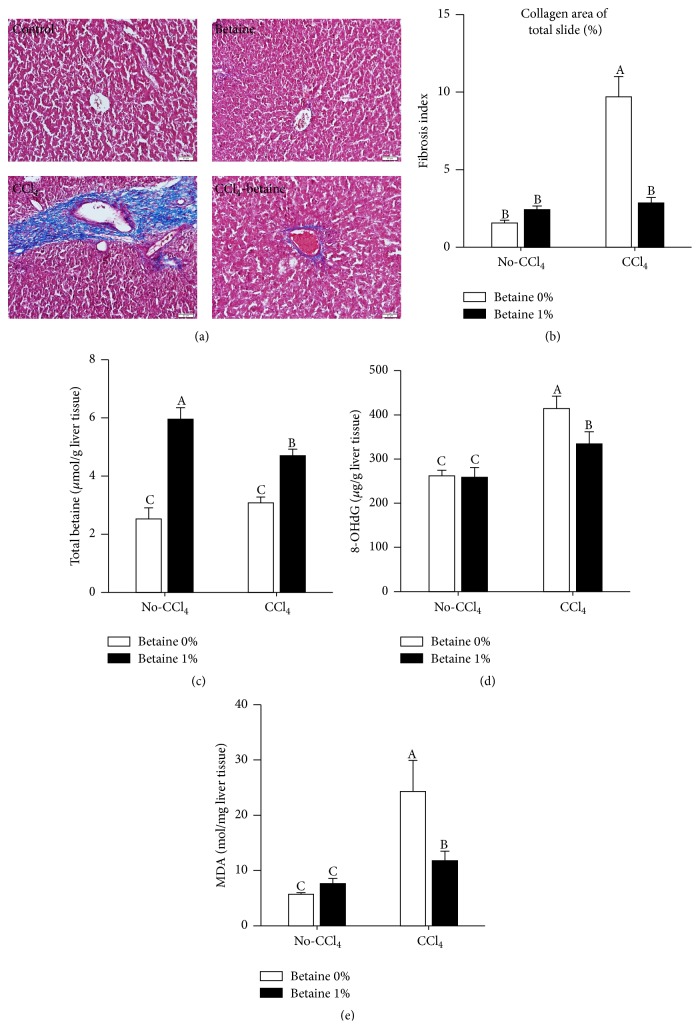
Betaine supplementation suppressed CCl_4_-induced liver fibrosis development. (a) Liver sections with Masson's staining. (b) Quantification of collagen area in Masson's staining by pixel calculation. (c) Concentration of total betaine in chicken liver. (d) Oxidative stress in chicken liver. (e) Chicken liver MDA concentrations after CCl_4_ challenge. Data were analyzed by two-way ANOVA (*n* = 6). Means with the same letter were not significantly different at *P* ≤ 0.05. 8-OHdG: 8-hydroxy-2-deoxyguanosine, MDA: malondialdehyde analysis.

We first examined the effects of betaine on liver fibrosis induced by CCl_4_. The abnormal collagen deposition is one of special characters of liver fibrosis [3]. Under Masson's trichrome staining, the markedly increased collagen deposition was discovered in the CCl_4_ group; addition of betaine with the CCl_4_ treatment significantly suppressed liver fibrosis in comparison with CCl_4_ group, and no fibrosis was found in the control or betaine groups (Figure 2(a)). The quantitative analysis showed that collagen deposition drastically decreased by exposure to betaine in comparison with CCl_4_ group (Figure 2(b)). The concentration of betaine in the liver with betaine group was significantly higher than CCl_4_-betaine group and control group (Figure 2(b)). To determine the changes of oxidative stress by betaine supplementation, the 8-OHdG and MDA levels of livers were measured to assess oxidative stress and lipid peroxidation; stimulation of CCl_4_ greatly increased 8-OHdG and MDA levels, and supplementation of betaine significantly decreased this response (Figures 2(d)-2(e)).

**Figure 3:**
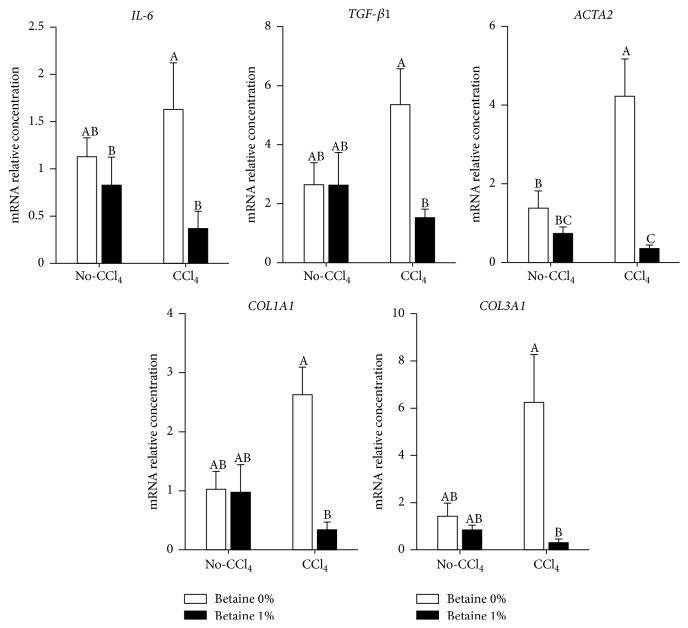
Effects of betaine on liver mRNA expressions of fibrogenesis related genes with CCl_4_-induced liver fibrosis (*n* = 6). Values were presented as the mean ± SEM. Data were analyzed by two-way ANOVA. Means with the same letter were not significantly different at *P* ≤ 0.05. Alpha smooth muscle actin (*ACTA2*), collagen type1-*α*1 (*COL1A1*), collagen type3-*α*1 (*COL3A1*), interleukin-6 (*IL-6*), and transforming growth factor-beta 1 (*TGF-β1*).

To quantitate the effects of betaine on CCl_4_-induced fibrogenesis, we examined the mRNA expression levels of* IL-6*,* TGF-β1*,* ACTA2*,* COL1A1*, and* COL3A1* in chicken livers; each of these genes represents different steps of liver fibrosis developing progress, whereas IL-6 represents the inflammation in respond to CCl_4_ stimulation in liver [36]; TGF-*β*1 as a key cytokine is involved in the pathogenesis of fibrosis in liver and the initiator of activated HSCs [16]; ACTA2 also known as alpha smooth muscle actin (*α*-SMA) is a marker of activated HSCs [37]; types I and III collagen mRNA (*COL1A1* and* COL3A1*) expression levels represent collagen productions in HSCs [38]. In our study, each gene's expression levels were increased by CCl_4_ injection, and the attenuated elevation of these gene expressions was discovered in betaine supplementation group (Figure 3). These results indicate that the antifibrotic effect of betaine might be provided by its antioxidant capacity. Furthermore, betaine might also affect the progression of liver fibrosis by suppressing activation of HSCs.

### 3.3. Effects of Betaine on Selected Liver Injury Biomarkers of Chicken

**Figure 4:**
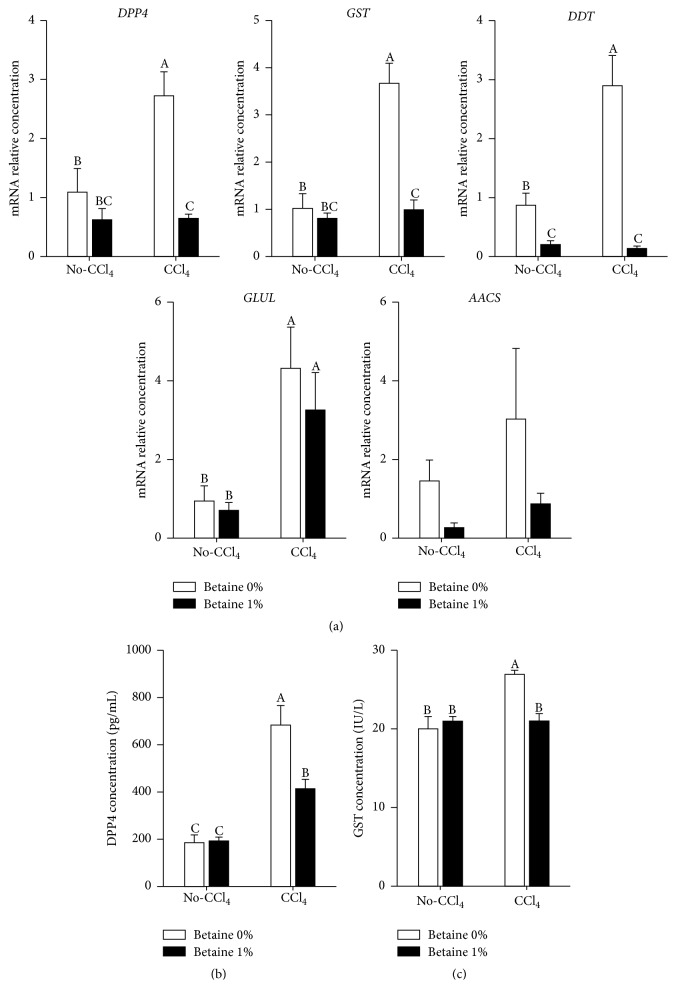
Effects of betaine on liver mRNA expressions of selected liver injury biomarkers and plasma DPP4 and GST concentrations with CCl_4_-induced liver fibrosis. (a) mRNA expressions of selected liver injury biomarkers. (b) Plasma DPP4 concentrations. (c) Plasma GST concentrations. Values were presented as the mean ± SEM. Data were analyzed by two-way ANOVA (*n* = 6). Means with the same letter were not significantly different at *P* ≤ 0.05. Acetoacetyl-CoA synthetase (*AACS*), D-dopachrome tautomerase (*DDT*), dipeptidyl-peptidase 4 (*DPP4*), glutamate synthase (*GLUL*), and glutathione S-transferase (*GST*).

As noted above, the mammalian liver injury biomarkers (AST and ALT) are inefficient in poultry [34, 35]. Unsurprisingly, current data showed CCl_4_-induced serious liver fibrosis did not significantly change the plasma AST and ALT levels in the present study (Table 2). For diagnosing liver injury levels of chickens, recently, our groups screened the chicken liver injury biomarkers through transcriptome and proteome analysis and found several useful biomarkers for liver fibrosis (unpublished observations). These biomarkers are acetoacetyl-CoA synthetase (AACS), dipeptidyl-peptidase 4 (DPP4), d-dopachrome tautomerase (DDT), glutamine synthetase (GLUL), and glutathione S-transferase (GST); particularly, the plasma DPP4 and GST levels were highly associated with CCl_4_-induced liver fibrosis. Here, we examined the mRNA expression levels of these chicken liver injury biomarkers and plasma DPP4 and GST concentrations in each group. CCl_4_ injection increased expressions of* DPP4*,* GST*,* DDT*, and* GLUL* (Figure 4(a)). We observed exposure of chickens to betaine could reverse these increments in* DPP4*,* GST*, and* DDT* expression levels (Figure 4(a)). Further, the plasma DPP4 and GST concentrations also followed the pattern of the mRNAs, DPP4 showed 1.6-fold decrease, and GST showed 1.3-fold decrease in CCl_4_-betaine group compared with CCl_4_ group (Figures 4(b)-4(c)). These results indicated the screening biomarkers from the current study were more effective than AST and ALT. Furthermore, the current results showed the antifibrotic effects of betaine were accompanied by decreasing gene expressions of screening biomarkers and plasma DPP4 and GST concentrations.

### 3.4. Betaine Affected Necrosis, Lipid Peroxidation, and Inflammation in Chicken Hepatocytes* In Vitro*

**Figure 5:**
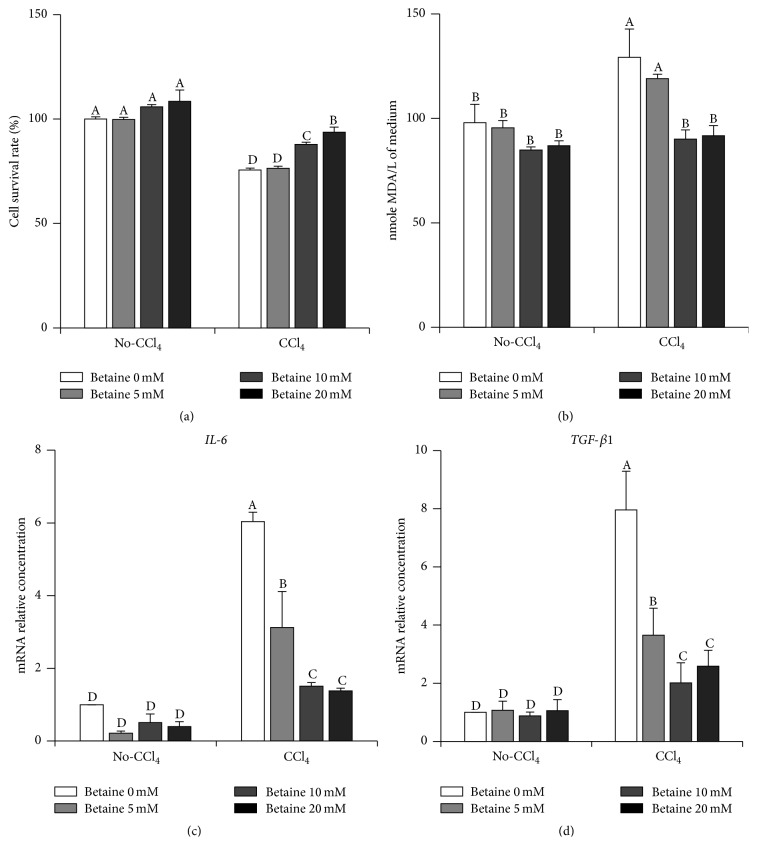
Effects of betaine on cell survival, lipid peroxidation, and proinflammation gene expression in chicken hepatocytes. (a) Cell survival after betaine and CCl_4_ treatments of hepatocytes for 20 hours (*n* = 4). (b) MDA levels of cell culture medium when cells were treated with CCl_4_ or betaine for 20 hours (*n* = 4). (c)* IL-6* mRNA levels and (d)* TGF-β1* mRNA levels in hepatocytes treated with CCl_4_ or betaine for 20 hours (*n* = 4). Values were presented as the mean ± SEM. Data were analyzed by two-way ANOVA. Means with the same letter were not significantly different at *P* ≤ 0.05. Malondialdehyde (MDA), interleukin-6 (*IL-6*), and transforming growth factor-beta 1 (*TGF-β1*).

We observed betaine treatment decreased the CCl_4_-stimulated 8-OHdG and MDA levels in the liver (Figures 2(d)-2(e)), suggesting that hepatic injury caused by CCl_4_ was alleviated by betaine, possibly because of its antioxidant properties. To confirm this speculation, we isolated chicken primary hepatocytes and examined the hepatocyte necrosis, lipid peroxidation, and inflammation within CCl_4_ and betaine treatments. In CCl_4_-treated hepatocytes, the cell survival ratio was significantly decreased. The cell survival ratio showed a reversed response in a dosage-dependent manner with betaine supplementation (Figure 5(a)). As* in vivo* data, the significantly elevated lipid peroxidation (measured by MDA concentration in the cell culture medium) was found in chicken primary hepatocytes exposed to CCl_4_, and this effect was also rescued by betaine treatment in a dosage-dependent manner (Figure 5(b)). We further examined the expression levels of* IL-6* and* TGF-β1* in chicken primary hepatocytes. Similar to the results of cell survival ratio and MDA levels, both* IL-6* and* TGF-β1* expression levels were increased by exposing CCl_4_, and these increments were also inhibited by betaine supplementation in a dosage-dependent manner (Figures 5(c)-5(d)). Taken together, these results suggested that the hepatoprotective effect of betaine is derived from suppressing CCl_4_-induced oxidative stress along with decreasing inflammation and activation of HSCs.

## 4. Discussion

Betaine is distributed broadly in plants, animals, and microorganisms and also rich in dietary sources [20]. In mammalian, the major physiologic functions of betaine are as an osmotic regulator and methyl group donor for the methionine-homocysteine cycle [23]. Previous studies indicate betaine could activate AMP-activated protein kinase to reduce lipid synthesis and fat accumulation in the liver to improve nonalcoholic fatty liver disease [39, 40]. In other liver injury studies, betaine also provides hepatoprotective effects against several hepatotoxicants such as CCl_4_, ethanol, lipopolysaccharide, *α*-naphthylisothiocyanate, and dimethyl nitrosamine [24–26, 41, 42]. Previous review of betaine concludes the betaine is safe at a daily intake of 9–15 g [20]. In addition, the nontoxicity of betaine (0–5% of the diet) has also been reported in subacute and subchronic rat studies [43]. Collectively, these studies suggest the supplementation of betaine could be a potential therapeutic strategy for liver diseases.

As noted above, HSCs have been suggested as the central factor of the overabundance of collagen and extracellular matrix production in liver fibrosis. In the progression of liver fibrosis, the activation of HSCs regulates the proliferation of HSCs. Previous studies demonstrate the proliferation and activation of HSCs are associated with the oxidative stress, which results from the increased production of lipid peroxidation and reactive oxygen species [3, 44]. Therefore, the antioxidant capacity is recognized as one of the critical therapeutic interventions of liver fibrosis.

In this study, we firstly examined that betaine could provide antifibrotic effects on CCl_4_-induced liver fibrosis in chickens. The histological detection provides the evidence of betaine suppressing the CCl_4_-induced collagen deposition.* In vitro* data confirmed that betaine could rescue the CCl_4_-induced hepatocyte necrosis. Previous study has indicated the supplementation of betaine could protect the lipopolysaccharide-induced hepatic necrosis by suppressing Kupffer cell activation and acting as an antioxidant [45]. For evaluating the antioxidant capacity of betaine, we examined the oxidative stress induced by CCl_4_, which showed a significant decreasing by treatment of betaine in both* in vivo* and* in vitro* studies. As far as the antioxidants exert anti-inflammatory activities [46], we also examined the anti-inflammatory ability of betaine by detecting the* IL-6* expression in liver and primary hepatocyte. Betaine supplementations significantly decreased the CCl_4_-stimulated IL-6 expression levels both* in vivo* and* in vitro*. As previous studies described, inflammatory cytokines and TGF-*β*1 showed interactions during the disease state. In liver fibrosis, TGF-*β*1 is one of the most crucial cytokines, which stimulates hepatic fibrogenesis through TGF-*β*1/Smad signaling pathway in activated HSCs. In activated HSCs, the upregulated TGF-*β* receptors induce the formation of Smad complex. This Smad complex then translocates into nucleus and regulates the transcription of collagens [16]. In our study, we showed that treatment with betaine suppressed the mRNA expression of TGF-*β*1, which was induced by CCl_4_ both* in vivo* and* in vitro*. Besides, the activation of HSCs and collagen synthesis were assessed by* ACTA2*,* COL1A1*, and* COL3A1* mRNA expression levels, which were also suppressed by betaine supplementation. Thus, we hypothesized that betaine may not only reduce the oxidative stress but probably also suppress the activation and functions of HSCs.

To verify whether betaine can directly alter the activation and functions of HSCs, we isolated chicken HSCs following Yata et al. [47]. The HSCs were stimulated with TGF-*β*1 and cotreated with betaine. We examined whether betaine directly suppressed the TGF-*β*1-dependents HSCs activation and collagen synthesis by detecting* ACTA2*,* DPP4*,* COL1A1*, and* COL3A1* mRNA expression levels and total collagen in the HSCs. As shown in supplementary Figure 1 (see Supplementary Material available online at http://dx.doi.org/10.1155/2015/725379), all these genes and total collagen contents were not altered by treatment of betaine, which indicates that the betaine cannot directly affect HSCs but provides the antioxidant capacity to reduce the oxidative stress on hepatocytes.

In human studies, elevated plasma levels of AST and ALT and increased ratio of AST/ALT (>1) work as biomarkers for liver injuries and alcoholic liver diseases [48–50]. However, the previous studies observed the inefficiency of AST and ALT in avian liver injury [34, 35], but some could detect the significant difference in avian liver injury [5]. Based on these inconsistent results, our previous study has suggested plasma DPP4 and GST concentration could serve as good potential biomarkers of chicken liver injury (unpublished data). As expected, we observed significant increasing in the plasma concentrations of DPP4 and GST by CCl_4_ stimulation, and supplementation of betaine alleviated these abnormal increments. There is a high association between serum DPP4 activity and liver injury in the rat [51]. Interestingly, previous studies demonstrate that DPP4 is only expressed in Ac-HSCs [52] and suggest inhibition of DPP4 activity could reduce hepatic fibrosis through suppression of HSCs proliferation and collagen synthesis [53]. These studies indicate the critical role of DPP4 in liver fibrosis progression, whereas DPP4-inhibitor has also been suggested to be a potential therapeutic agent for liver fibrosis [53]. In the present study, we firstly found the significant amelioration of liver fibrosis was accompanied with the decrement of DPP4 hepatic gene expression and plasma DPP4 concentrations when treated with betaine. Previous study has described that DPP4-inhibitor could suppress TGF-*β*1 signaling pathways (Smad2/3 and ERK1/2), which regulate the collagens synthesis and HSCs proliferation [16]. In our study, we also found that betaine treatment suppressed expression of TGF-*β*1, activation of HSCs, and collagen formation in the liver. Collectively, the results of DPP4 provided evidence that betaine treatment suppressed the activation of HSCs.

The other liver injury biomarker, GST, can function through catalyzing the conjugation of the antioxidant, glutathione, which detoxifies endogenous compounds including peroxidised lipids and xenobiotics [54, 55]. In the present study, hepatic GST gene expression and plasma concentrations of GST showed increments under CCl_4_ stimulation, and these increments were reversed by betaine supplementation. The elevated plasma GST concentration in response to CCl_4_ stimulation may represent a defense mechanism in the hepatocytes to combat lipid peroxidation by producing more glutathione. These effects confirmed the antioxidant capacity of betaine in coping with CCl_4_-induced oxidative stress.

## 5. Conclusion

**Figure 6:**
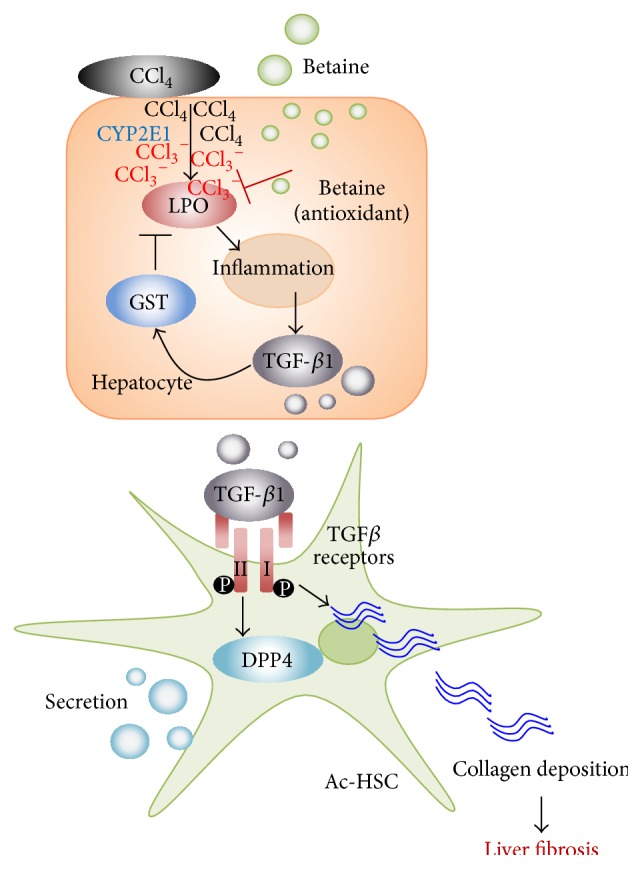
Antioxidant capacity of betaine and its role in CCl_4_-induced liver fibrosis. Under CCl_4_ stimulation, cytochrome P450 2E1 (CYP2E1) transforms CCl_4_ to CCl_3_^−^. CCl_3_^−^ is a free radical, which induces lipid peroxidation (LPO) and causes oxidative stress. The cellular elevated oxidative stress induces secretion of inflammatory cytokines, including interleukin-6 (IL-6). Inflammation cytokines upregulate glutathione S-transferase (GST) and transforming growth factor-beta 1 (TGF-*β*1). GST as an antioxidant provides the self-protective mechanism to eliminate free radicals and LPO. Hepatocyte secreted TGF-*β*1 activates hepatic stellate cells (Ac-HSCs) through binding with TGF-*β* receptors. Further, the activated HSCs process fibrogenesis and synthesize collagens, finally leading to liver fibrosis. Moreover, Ac-HSCs generate and secret the dipeptidyl-peptidase 4 (DPP4), which leads us to detect the increasing plasma DPP4 concentrations in liver fibrosis chicken. Betaine provides the antioxidant capacity to alleviate the oxidative stress, which suppresses the effects of CCl_4_ in the very beginning.

In summary, we demonstrated the treatment with betaine evidently suppressed liver fibrosis along with alleviation of lipid peroxidation, inflammation, and activation of HSCs. These beneficial effects of betaine might associate with its antioxidant ability, which is against CCl_4_-induced cell necrosis and lipid peroxidation. Based on the results, we hypothesized a possible mechanism scheme of betaine against liver fibrosis in Figure 6. Because the chicken has potential to be a human liver disease model and based on the significant antifibrotic effects of betaine in CCl_4_-induced liver fibrosis, we suggested that betaine may represent a potent therapeutic agent for liver fibrosis.

## Supplementary Material

To clarify whether betaine can directly suppress the activation of HSCs, we isolated the primary chicken HSCs, stimulated with TGF-β1 and co-treated with different concentrations of betaine. First, we detected the total collagen concentration in the HSCs culture medium, the results showed betaine supplementation did not suppress TGF-β1-stimualted collagen secretion (Figure S1 a). Furthermore, to confirm the effect of betaine on TGF–β1-activated HSCs, we used real-rime PCR to detect the expression of HSCs activation and collagen synthesis related genes (ACTA2, DPP4, COL1A1 and COL3A1). The result of collagen content in the culture medium indicated that the betaine supplementation cannot suppressed the TGF-β1-evelvated gene expressions. These results suggested treatments of betaine cannot directly suppress TGF-β1-stimulated HSCs activation and collagen synthesis.

## Figures and Tables

**Table 1:** List of primer sequences for quantitative real-time PCR.

Gene symbol	Primer sequences 5′-3′	Anneal temp. (°C)	GenBank accession number	Product size (bp)
IL-6	F: CTCCTCGCCAATCTGAAGTC	60	NM_204628.1	164
	R: GGATTGTGCCCGAACTAAAA			

TGF-*β*1	F: AGCCACAGCATCTTCTTCGT	58.4	JQ423909.1	162
	R: ATTGCCGTAACCCTGGTACA			

ACTA2	F: CACCCAACTCTGCTGACTGA	61.4	NM_001031229.1	176
	R: ACACCATCCCCAGAGTCAAG			

COL1A1	F: GAACCCCAAGGAGAAGAAGC	58.4	XM_423116.4	167
	R: TCTTGCAGTGGTAGGTGACG			

COL3A1	F: AGGCTGAAGGAAACAGCAAA	58.4	NM_205380.2	102
	R: TGCGCGTTCTGTATTCAAAG			

DPP4	F: GGTGGGCACACTTTCCTAAA	60	NM_001031255.1	101
	R: TTGAGCATTCAGGCAACAAG			

GST	F: ACCTGCTTCAAAAGCTGGAA	58.4	NM_205412.1	130
	R: GTTAGCCAGGACTGCCAGAG			

DDT	F: TTGCTGTTGCACTTCACTCC	58.4	NM_001030667.1	152
	R: GAAGCCAGTGCATGTTCTCA			

GLUL	F: GCTCAGTGGGGAAGACTCAG	58.4	NM_205493.1	177
	R: GGTCCAGAACGTGGTGAAGT			

AACS	F: CCAGCTAATGCGTGCTGATA	63	NM_001006184.1	162
	R: TTGACTGCTTGTTGCTGTCC			

ACTB	F: GTGATGGACTCTGGTGATGG	62	NM_205518.1	151
	R: TGGTGAAGCTGTAGCCTCTC			

IL-6, interleukin-6; TGF-*β*1, transforming growth factor-*β*1; ACTA2, *α*-smooth muscle actin; COL1A1, collagen type 1-*α*1; COL3A1, collagen type 3-*α*1; DPP4, dipeptidyl-peptidase 4; GST, glutathione S-transferase; DDT, d-dopachrome tautomerase; GLUL, glutamine synthetase; AACS, acetoacetyl-CoA synthase; and ACTB, *β*-actin.

**Table 2:** Characteristic features of the experimental groups.

	Control	Betaine	CCl_4_	CCl_4_-betaine
Body weights (g)	60.766 ± 1.030^a^	58.515 ± 0.626^a^	61.890 ± 0.758^a^	54.146 ± 0.838^b^
Relative liver weight (%)	2.804 ± 0.090^b^	3.177 ± 0.052^b^	2.670 ± 0.033^b^	3.4122 ± 0.097^a^

Plasma parameters:				
AST (IU/L)	81.364 ± 8.635	63.372 ± 6.023	84.310 ± 11.500	78.266 ± 4.017
ALT (IU/L)	3.492 ± 0.943	3.543 ± 0.534	2.968 ± 0.810	2.729 ± 0.398

Values were presented as the mean ± SEM. Data were analyzed by two-way ANOVA (*n* = 6). Means with the same letter were not significantly different (*P* ≥ 0.05).
