# Supplementary material for: Alleviation of Carbon-Tetrachloride-Induced Liver Injury and Fibrosis by Betaine Supplementation in Chickens
Source: Evid Based Complement Alternat Med. 2015 Sep 27;2015:725379. doi: 10.1155/2015/725379 (PMC4600548; doi:10.1155/2015/725379)
Supplement: Supplementary file 1 — To clarify whether betaine can directly suppress the activation of HSCs, we isolated the primary chicken HSCs, stimulated with TGF-β1 and co-treated with different concentrations of betaine. First, we detected the total collagen concentration in the HSCs culture medium, the results showed betaine supplementation did not suppress TGF-β1-stimualted collagen secretion (Figure S1 a). Furthermore, to confirm the effect of betaine on TGF–β1-activated HSCs, we used real-rime PCR to detect the expression of HSCs activation and collagen synthesis related genes (ACTA2, DPP4, COL1A1 and COL3A1). The result of collagen content in the culture medium indicated that the betaine supplementation cannot suppressed the TGF-β1-evelvated gene expressions. These results suggested treatments of betaine cannot directly suppress TGF-β1-stimulated HSCs activation and collagen synthesis. [file 725379.f1.docx]

2cig. 2c 2cig. 2c**Alleviation of carbon-tetrachloride-induced liver injury and fibrosis by betaine supplementation in chickens**

Meng-Tsz Tsai^1^, Ching-Yi Chen^1^, Yu-Hui Pan^1^, Siou-Huei Wang^1^, Harry J. Mersmann^1^, Shih-Torng Ding^1, 2^

^1^*Department of Animal Science and Technology, National Taiwan University, No. 50, Ln. 155, Sec. 3, Keelung Rd., Taipei City 106, Taiwan, R.O.C.*

^2^ *Institute of Biotechnology, National Taiwan University, No. 81, Chang-Xing St., Taipei 106, Taiwan, R.O.C.*

Correspondence should be addressed to Shih-Torng Ding; [sding@ntu.edu.tw](mailto:sding@ntu.edu.tw)

**
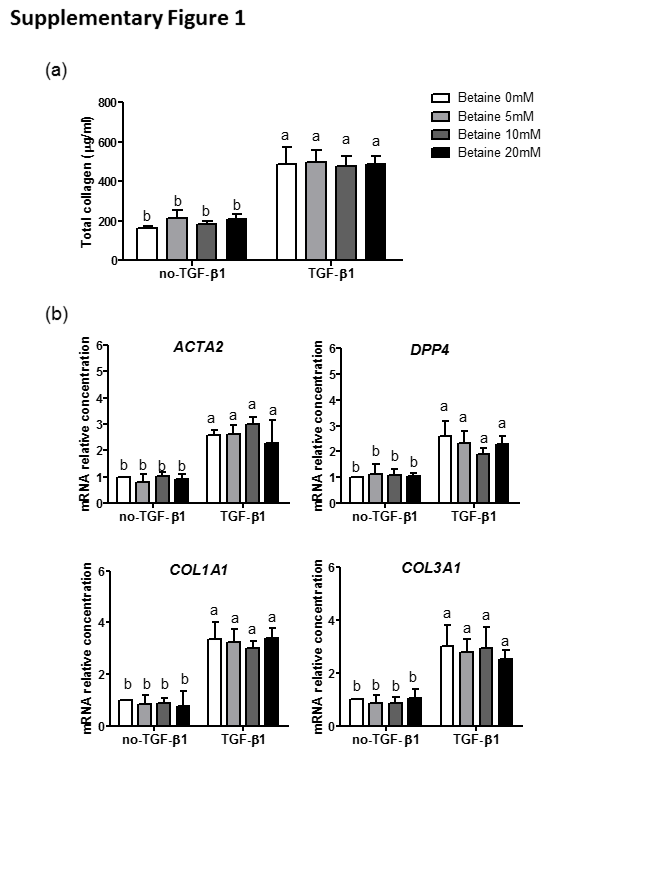
Supplementary Figure 1. Treatment of betaine cannot suppress TGF-β1-stimulated HSCs activation and collagen synthesis**. (a) The level of total collagen in the HSCs cultured mediums after stimulated with TGF-β1 and treated with betaine for 24 hours. (b) Effects of betaine on HSCs activation and fibrogenesis related genes after TGF-β1 and betaine treatment. Values were presented as the mean ± SEM (n=3). Data were analyzed by two-way ANOVA. Means with the same letter were not significantly different at *p*≤0.05. Alpha-smooth muscle actin (*ACTA2*), Collagen type1-α1 (*COL1A1*), Collagen type3-α1 (*COL3A1*) and Transforming growth factor-beta (*TGF-β1*).
